# Towards understanding the influence of porosity on mechanical and fracture behaviour of quasi-brittle materials: experiments and modelling

**DOI:** 10.1007/s10704-017-0181-7

**Published:** 2017-01-12

**Authors:** Dong Liu, Branko Šavija, Gillian E. Smith, Peter E. J. Flewitt, Tristan Lowe, Erik Schlangen

**Affiliations:** 1 School of Physics, University of Bristol, Bristol, BS8 1TL UK; 2 Microlab, Faculty of Civil Engineering and Geosciences, Delft University of Technology, 2628 CN Delft, The Netherlands; 3 HH Wills Physics Laboratory, School of Physics, University of Bristol, Bristol, BS8 1TL UK; 4 School of Materials, University of Manchester, Manchester, M13 9PL UK; 5 Present Address: Department of Materials, University of Oxford, Parks Road, Oxford, OX1 3PH UK

**Keywords:** Quasi-brittle materials, Porosity, Strength, Microstructure

## Abstract

In this work, porosity-property relationships of quasi-brittle materials are explored through a combined experimental and numerical approach. In the experimental part, hemihyrate gypsum plaster powder ($\hbox {CaSO}_{4}\cdot 1/2\hbox {H}_{2}\hbox {O}$) and expanded spherical polystyrene beads (1.5–2.0 mm dia.) have been mixed to form a model material with controlled additions of porosity. The expanded polystyrene beads represent pores within the bulk due to their light weight and low strength compared with plaster. Varying the addition of infill allows the production of a material with different percentages of porosity: 0, 10, 20, 30 and 31 vol%. The size and location of these pores have been characterised by 3D X-ray computed tomography. Beams of the size of $20 \times 20 \times 150$ mm were cast and loaded under four-point bending to obtain the mechanical characteristics of each porosity level. The elastic modulus and flexural strength are found to decrease with increased porosity. Fractography studies have been undertaken to identify the role of the pores on the fracture path. Based on the known porosity, a 3D model of each microstructure has been built and the deformation and fracture was computed using a lattice-based multi-scale finite element model. This model predicted similar trends as the experimental results and was able to quantify the fractured sites. The results from this model material experimental data and the lattice model predictions are discussed with respect to the role of porosity on the deformation and fracture of quasi-brittle materials.

## Introduction

Damage and fracture in materials such as ceramics, rock, concrete and nuclear graphites are usually complicated by the existence of pores and aggregates with various sizes and geometry (Bazant and Planas 1997; Rice 1984). Many of these materials can be considered as perfectly brittle with their quasi-brittle behaviour arising from the presence of defects such as micro-cracks, pores (Hillerborg et al. 1976) aggregate or filler particles (Moskovic 2014). The important and extensive opportunities and need for these porous materials in the field of medicine, energy, and aerospace are illustrative of the imperative driving forces for understanding the effect of microstructural porosity on the mechanical material properties. Porosity can arise either as a consequence of the production process for the material, such as in the case of concrete (Kendall et al. 1983), or be due to in-service ageing and deterioration, for example in the Gilsocarbon graphite contained in the core of advanced gas-cooled nuclear reactors subject to radiolytic oxidation (Šavija et al. 2016). In the latter case, in particular, it is essential to be able to predict the mechanical properties as the porosity evolves in the graphite to ensure the structural integrity of the reactor core. In practice the mechanical and fracture characteristics are a consequence of the influence of several parameters. To obtain a mechanistic understanding, a simplified model material with controlled additions of porosity was manufactured, characterised, tested and modelled in the present study.

It has been recognised that in addition to changing the response of a linear elastic material to quasi-brittle, increased porosity affects the mechanical properties of materials such as tensile, compressive and flexural strength (Birchall et al. 1981), elastic modulus, fracture energy and fracture toughness (Chang et al. 2000; Rice 1984, 1989). The porosity dependence of these parameters follows a similar trend under tensile loading although Young’s modulus as a function of porosity usually shows less variation in comparison to strength. When tested under compressive loading, the greatest changes in strength and hardness are observed. (Duckworth 1953) first described the relationship between porosity and compressive strength in ceramics and this was subsequently tested experimentally by Ryshkewitch (1953) on porous sintered alumina and yttria stabilized zirconia. Typically, the strength, $$\sigma $$, at a certain porosity level (in volume), $$P_V $$, is related to pore-free strength, $$\sigma _0 $$, by:

1 $$\begin{aligned} \sigma =\sigma _0 e^{-b_\sigma P_V } \end{aligned}$$

where $b_{\sigma }$ is an empirical constant which can be extracted from the slope of a semi-logarithmic plot of the strength–porosity curve. In the case of randomly distributed, isolated, spherical pores, the strength constant $b_\sigma$ is around 2.5–2.9 for glass (2–12% porosity) and 2.7 (0–50% porosity) to 4 (10–37% porosity) (Knudsen 1959). This relationship was then extended to elastic modulus, *E*, by Spriggs (1961) for porous polycrystalline refractory materials:

2 $$\begin{aligned} E=E_0 e^{-b_E P_V } \end{aligned}$$

where $$E_{0}$$ is the elastic modulus of the pore-free material. The modulus constant $$b_E $$ varies from 2.1 for glass to 2.8 for alumina (10 to 37% porosity). Since then, other similar empirical / semi-empirical or analytically-based models have been proposed for brittle solids, such as the elastic modulus model from (Phani and Niyogi 1987) and (Hasselman 1962).

The factors that classify a material as quasi-brittle have been described previously for example by Hodgkins et al. (2012) and Moskovic (2014) Linear elastic and quasi-brittle fracture are distinguished by the damage process introduced in the material. For a linear elastic material the load-displacement (stress–strain) response in tension is a continuous linear rise to peak load followed by prompt fracture. The load-displacement curve for a quasi-brittle material is characterised by the following: (1) a non-linear rise to peak load; (2) post-peak there is a progressive loss of load-carrying capacity. Non-linearity in the load-displacement curve is associated with the formation of distributed microcracks which accumulate close to peak load to form a macrocrack. The overall response of the material is related for example to the amount of porosity in the material, since they promote the formation of microcracks.

**Fig. 1:**
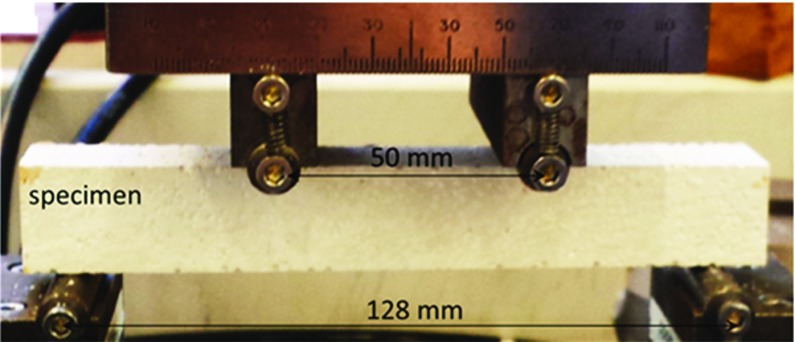
Experimental setup for four-point bending test of $20 \times 20 \times 150$ mm beams

In the pursuit of understanding the relationship between porosity, microstructure and mechanical properties of inherently brittle materials, particular effort has been devoted to the investigation of pore design and optimisation in porous hydroxyapatite (HA) and other related calcium phosphate ceramics considered for bone replacement. The presence of pores in this material is a critical requirement for osteoconduction while this introduces a corresponding reduction in strength (Tampieri et al. 2001). Le Huec et al. (1995) studied the compressive strength of HA samples with open porosity from 20 to 60% by volume with the sizes ranging from 5 to 400 $\upmu \hbox {m}$, and a polynomial equation was derived to predict the mechanical strength of this material for the optimisation of pore size and volume to balance the requirement for the colonization process and high strength. Similar work e.g. by Liu (1997) testing this type of ceramic with up to 78% porosity have consistently found that, for the same pore volume fraction, smaller macro-pores exhibited a higher compressive strength, and that the strength can be correlated exponentially with porosity volume with a correlation factor greater than 0.96. On the other hand, (Birchall et al. 1981) proposed that the existence of large pores is one of the limiting factors for the tensile and flexural strength of brittle materials, such as hydraulic cements. For the study of HA materials, the main approach to synthesize porous model materials is by using a polyurethane foam or net coated with HA to create sponge or cross-type of interlinking pores (Chang et al. 2000) and some other approaches such as water-based sol–gel (Liu et al. 2001). In these studies, pores are usually characterised by mercury porosimetry, optical and scanning electron microscopy combined with density measurements.

For a given brittle material, pores act as stress concentration sites and accentuate failure that initiated from another source and also promote failure under loading by introducing microcracks. Hence, an increase in pore volume reduces the elastic modulus, fracture energy, fracture toughness and the strength of the material. The shapes, sizes and locations of pores are all parameters affecting the deformation and fracture properties (Chandler et al. 2002). Detailed knowledge of these parameters is required to study the effect of pore distribution on mechanical properties. In the present work, we study isometrically isolated closed spherical pores and their effect on the mechanical properties by combining experimental tests and numerical modelling. We have developed a brittle model material with isolated pores randomly distributed and their size and geometry controlled. The aim is to reduce the number of controlling factors to facilitate studying the effect of porosity on fracture, in a systematic way. The materials investigated have a network of pores with the total pore volume varying from about 5% to about 30%. To explore the influence of porosity on fracture, it is essential to have well-characterised, three dimensional information about the material. Therefore, three dimensional X-ray computed tomography has been adopted in the current study to measure the pore fraction and the pore size distribution.

## Experiments and modelling

### Materials and techniques

A high strength hemihydrate plaster ($\hbox {CaSO}_{4}\cdot 1/2\hbox {H}_{2}\hbox {O}$) was produced from high purity gypsum mineral, which produced a very hard cast. The plaster to water ratio was controlled around 2.86:1 (2.86 kg/l). Expanded polystyrene beads (EPS), typically between 1.5 and 2.0 mm in diameter (nominal density 23 $\hbox {kg/m}^{3})$ were selected to mix with the plaster powder prior to adding water. Dry mixing ensured that the polystyrene agglomerates did not break up and were distributed randomly within the powder. The stock was mixed with a special plaster mixing power tool rotated at 250 rpm for 5 mins before being poured into a cast iron mould. This produced a cube with a dimension of $150 \times 150 \times 150$ mm that was left to dry for 60 days in air at room temperature. These cubes were then cut using a slow speed band saw into beams of $20 \times 20 \times 150$ mm. These beams were tested in four-point bending with an Instron 8872 Servo-Hydraulic Fatigue Testing machine with a 10 kN load cell. The setup is shown in Fig. 1. The outer support span was 128 mm and the loading span 50 mm with roller diameter of 7 mm. The loading process was displacement-controlled with a loading speed of 0.05 mm/min. Three specimens at each porosity level have been tested and the elastic modulus and flexural strength derived from the load-displacement curve.

The volume of the polystyrene beads was measured in a graduated cylinder; however, due to spaces between touching spherical polystyrene beads, this measuring system provides only an approximation of the volume of added beads. To overcome the uncertainty associated with the addition volume of pores in each sample, the actual porosity of each batch of sample was determined by 3D X-ray computed tomography using a Nikon Metrology 225/320 kV Custom Bay system (Manchester University, UK) with a voxel size of $16.0 \times 16.0 \times 16.0$ $\upmu \hbox {m}$ on a cube sample of $20 \times 20 \times 20$ mm. Six specimens, each with a different levels of added porosity (including zero) were made, and five were characterised using X-ray computed tomography.

### Modelling

#### Microstructure

The microstructure of the gypsum model material consists of air bubbles and expanded polystyrene beads as surrogate pores within a plaster matrix. Models were initiated as plaster bricks in which spherical pores were then embedded. The air bubbles were not modelled explicitly as experimental specimens without any addition of expanded polystyrene beads were made. Air bubbles were also present in these specimens, which were tested to measure the mechanical properties of the plaster that were input for the numerical simulations. In this way, the contribution of the air bubbles to the fracture strength of the plaster was accommodated in the modelling. The results presented relate to microstructural models matching the size of the test specimens ($20 \times 20 \times 150$ mm). The symmetry of the model space allowed four different fracture simulations to be performed with the same pores (volume fraction, size and distribution) but different stress axes.

**Fig. 2:**
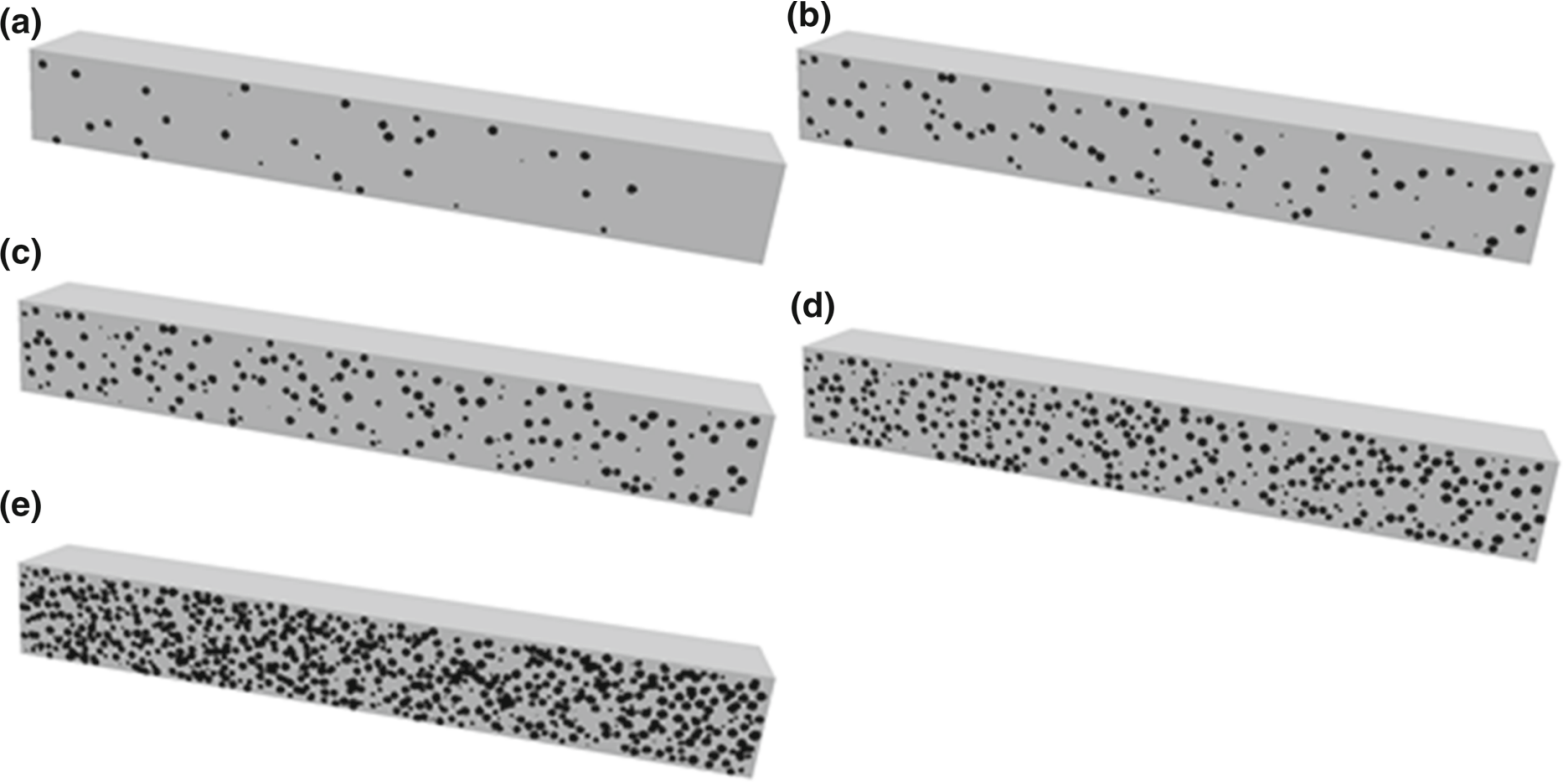
Computer generated microstructures of beam specimens used in mechanical simulations: **a** 2 vol% porosity; **b** 5 vol% porosity; **c** 10 vol% porosity; **d** 20 vol% porosity; and **e** 40 vol% porosity. *Grey colour* indicates the solid part, while *black* indicates the porosity

The microstructure of each model was defined by the centre co-ordinates and radius for each spherical pore within the model space. Model pores were generated successively; each having a diameter chosen randomly from the diameter range of the expanded polystyrene beads and its volume added to the total porosity. As each pore was created, random co-ordinates were assigned within the model space for the centre. Each new pore was tested to ensure it did not overlap any previous pore and failures were rejected. This process was repeated until the target porosity was achieved. For low volumes of pores, greater variability in pore positions was possible but for 30–40% porosity the constraints control positioning and little variability was achievable. Created microstructures are shown in Fig. 2. The geometric formulation of each model was mapped on to a three dimensional rectangular grid, with each voxel set to zero or one, determined by whether its centre lay within a pore or within the matrix. The voxel size (0.25 mm) was selected to be appropriate for input to the analysis software and a multi-scale approach to the deformation and fracture simulation (described in detail in Sect. 2.2.2).

#### Mechanical

For the analysis of deformation and fracture behaviour of gypsum plaster including various additions of porosity, a lattice type model is used. Lattice models have been used in the past to simulate deformation and fracture in quasi-brittle materials such as concrete (Bolander et al. 2000; Schlangen and Garboczi 1997; Schlangen and Qian 2009), nuclear graphite (Šavija et al. 2016; Smith et al. 2013), and rock (Asahina et al. 2014; Sands 2016). Unlike conventional approaches based on continuum mechanics, the material is discretized as a set of two-node (spring, truss, or beam) elements which can transfer forces in lattice models. Cracking is typically simulated by damaging these elements. In the model used for the present simulations, a regular cubical lattice grid of Timoshenko beam elements is used for discretization of the gypsum plaster material. As the load is applied on the mesh, a linear elastic analysis is performed by calculating the response of the lattice mesh. A single element with the highest stress-to-strength ratio is identified and removed from the mesh when it exceeds the fracture strength. Therefore, the model uses a brittle failure criterion on the local (i.e. element) scale. Removal of an element introduces a small crack in the mesh. Then the analysis is repeated with the updated geometry, and these steps are repeated until a prescribed criterion (e.g. deformation or load) is reached. Even though local behaviour of each element is brittle, quasi-brittle behaviour of complex materials can be simulated this way in a straightforward manner.

**Fig. 3:**
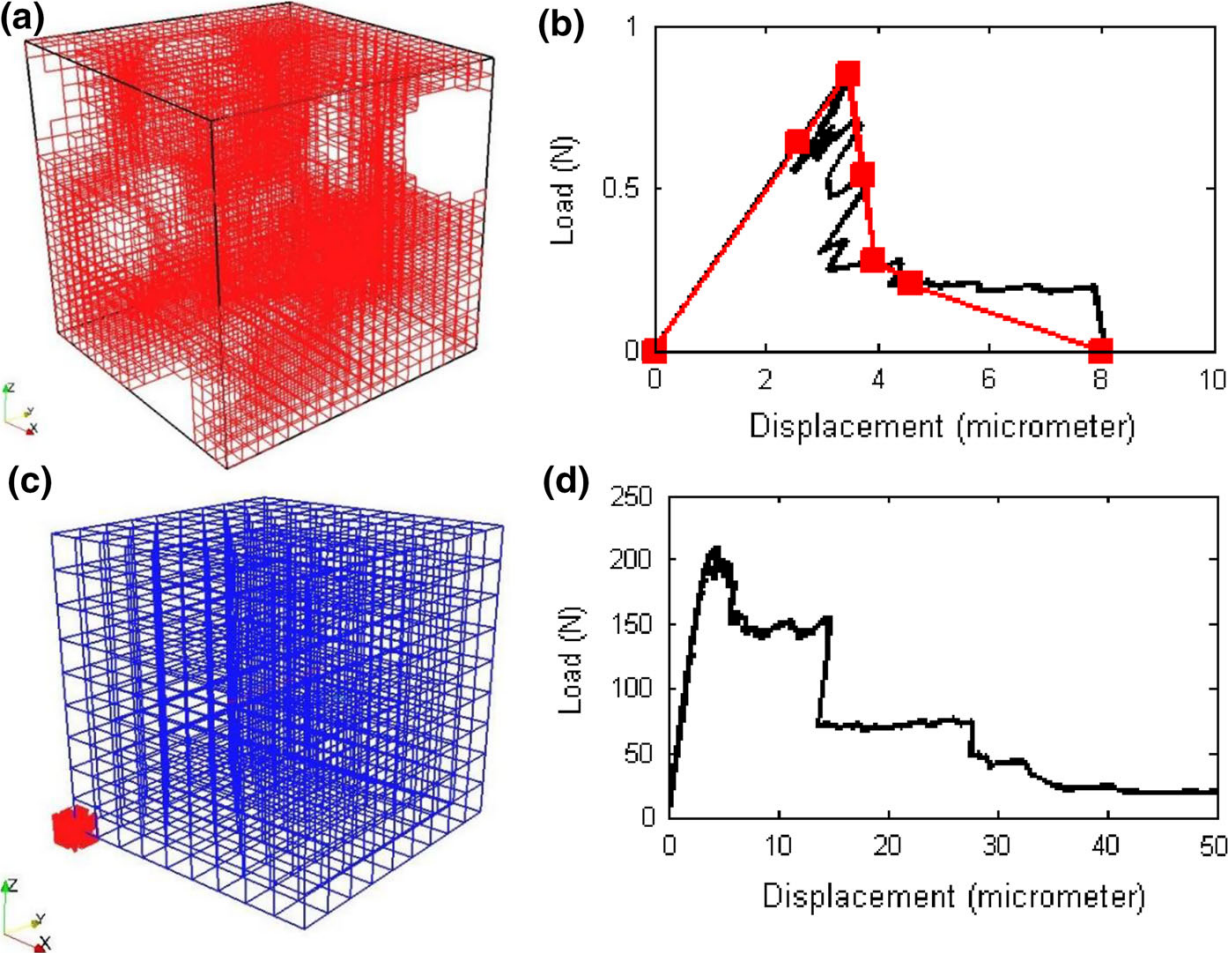
Multi-scale modelling procedure: **a** a small cube, $20 \times 20 \times 20$ voxels; **b** load-displacement curve (*black*), which is the outcome of a small-scale simulation. The *red curve* is a schematisation of the *black* one and is a multi-linear curve with six segments with points taken at (1) origin; (2) first micro-cracks; (3) peak load; (4) first point in response for which load is <75% of the peak; (5) first point in response for which the load is <50% of the peak; (6) first point in response for which the load is <25% of the peak; (7) point at which load is zero.; **c** detailed mesh shows a small cube ($20 \times 20 \times 20$ voxels) in *red* at its location within the large cube; **d** example load-displacement curve obtained from the full-scale simulation

In the simulations applied to the model porous gypsum plaster, it is important that the fine microstructural features (i.e. pores) are captured by the model. However, due to the computational demand, this is impossible to achieve using a single length-scale model. Therefore, in this paper, a multi-scale modelling approach, as previously described by Šavija et al. (2016), Smith et al. (2013) (and illustrated in Fig. 3) was used. For clarity, the modelling approach will be described in full. The first step is to divide the large microstructure (such are those shown in Fig. 1) into smaller cubes. Then, a simulated uniaxial tensile test is performed on each of these small cubes, referred to as small scale simulations. In these small scale simulations, all beam elements locally have linear elastic-ideally brittle behaviour, as described previously. The small scale simulations result in a number of stress/strain curves, which are then schematized as multi-linear. Multi-linear curves are then used as constitutive relations for beam elements in the larger cube (this we will refer to as full scale simulations). In the full-scale simulations, the model specimen is subjected to four-point bending, the same as in the experiments. In these simulations, again, the local element behaviour is not ideally brittle: each element was assigned a multi-linear constitutive relation according to the small scale simulations. Consequently, an element is not removed in each analysis step, but if an element reached a maximum stress its stiffness would change according to its specific constitutive relation. This essentially means that this element will adopt the properties of the next point in the local multi-linear constitutive relation. It should be noted, however, that multi-scale methods based on discretization of the larger domain by non-overlapping small-scale domains (SSDs), such as the multi-scale lattice model used herein, can lead to occurrence of cracks which are discontinuous across SSD boundaries. This issue has been recently resolved by Sencu et al. (2016), who used the same number of overlapping SSDs, thereby resolving the issue of crack discontinuity. This is outside of the scope of the current study, and will be a part of future research.

In the present work, a fracture criterion based on the tensile stress in beams is adopted. Normal force and bending moments in lattice beam elements are both taken into account by the following general relation:

3 $$\begin{aligned} \sigma =\alpha _N \frac{N}{A}+\alpha _M \frac{\max (M_X ,M_Y )}{W} \end{aligned}$$

where *A* is the beam cross-sectional area, *W* the cross sectional moment of resistance, and are the normal force influence factor and the bending influence factor. Their values are most commonly adopted as 1.0 and 0.05, respectively. These values have also been adopted herein. The critical stress value was selected based on the measurements performed on the 0% porosity specimen, as explained below.

**Fig. 4:**
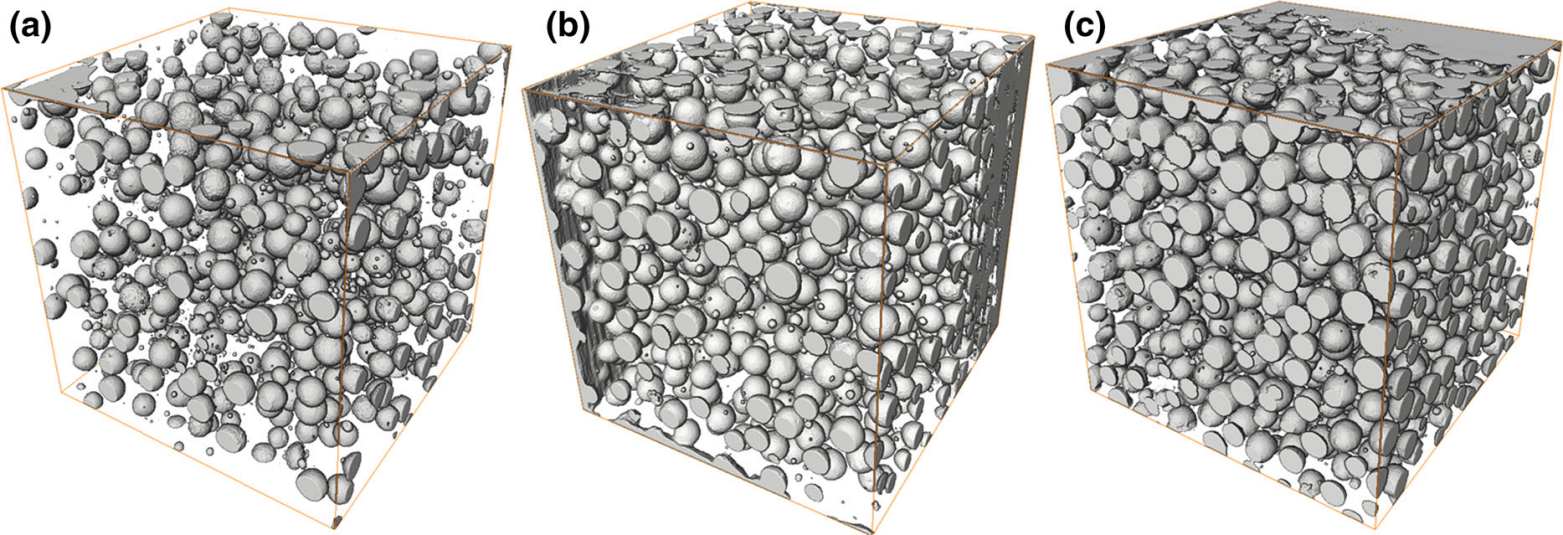
The reconstructed tomographic images of the material with total porosity of **a** 12.9 vol%; **b** 22.6 vol% and **c** 30.4 vol%. The *grey coloured* solid particles represent the added expanded polystyrene beads. Small pores are air bubbles produced during manufacture

Four point bend geometry test experiments were simulated using the described multi-scale modelling concept. The model needs very few parameters as input: the microstructure and the mechanical properties. First, as input, computer generated microstructures shown in Fig. 2 were used. The microstructures were generated such that they cover the range of porosities which were experimentally studied. For the mechanical properties (elastic modulus and tensile strength) of lattice elements, the modulus and fracture strength data measured from the 0% porosity specimen were used (Fig. 7). These properties were calibrated by simulating a 0% specimen to obtain correct input values. The elastic modulus was therefore set to 14.69 GPa (measured experimentally), and the tensile strength to 17.4 MPa ($\sim$60% of the measured bending strength, which was 29 MPa). It should be noted that, as described in Sect. 2.2.1, air bubbles (introduced in the material as a consequence of the mixing process) were not modelled explicitly: since they were present in the nominally pore free specimen (i.e. the specimen without EPS addition), their contribution is implicitly included in the model via mechanical properties. This is because, as described further in Sect. 3.1, the volume, size, and distribution of these air bubbles remains relatively constant in all specimens.

## Results

### Microstructure

Cube specimens ($20\times 20\times 20$ mm) with five different added porosity levels have been characterised by X-ray computed tomography. Three typical reconstructed images are shown in Fig. 4 with 12.9, 22.6 and 30.4 vol%, respectively. In particular, for the zero added porosity, the cube is not pore-free since 0.5 vol% porosity was measured. The micro-metre scale air bubbles contained in the zero-EPS porosity specimen and this could not be avoided after several attempts of varying the manufacture procedures.

**Fig. 5:**
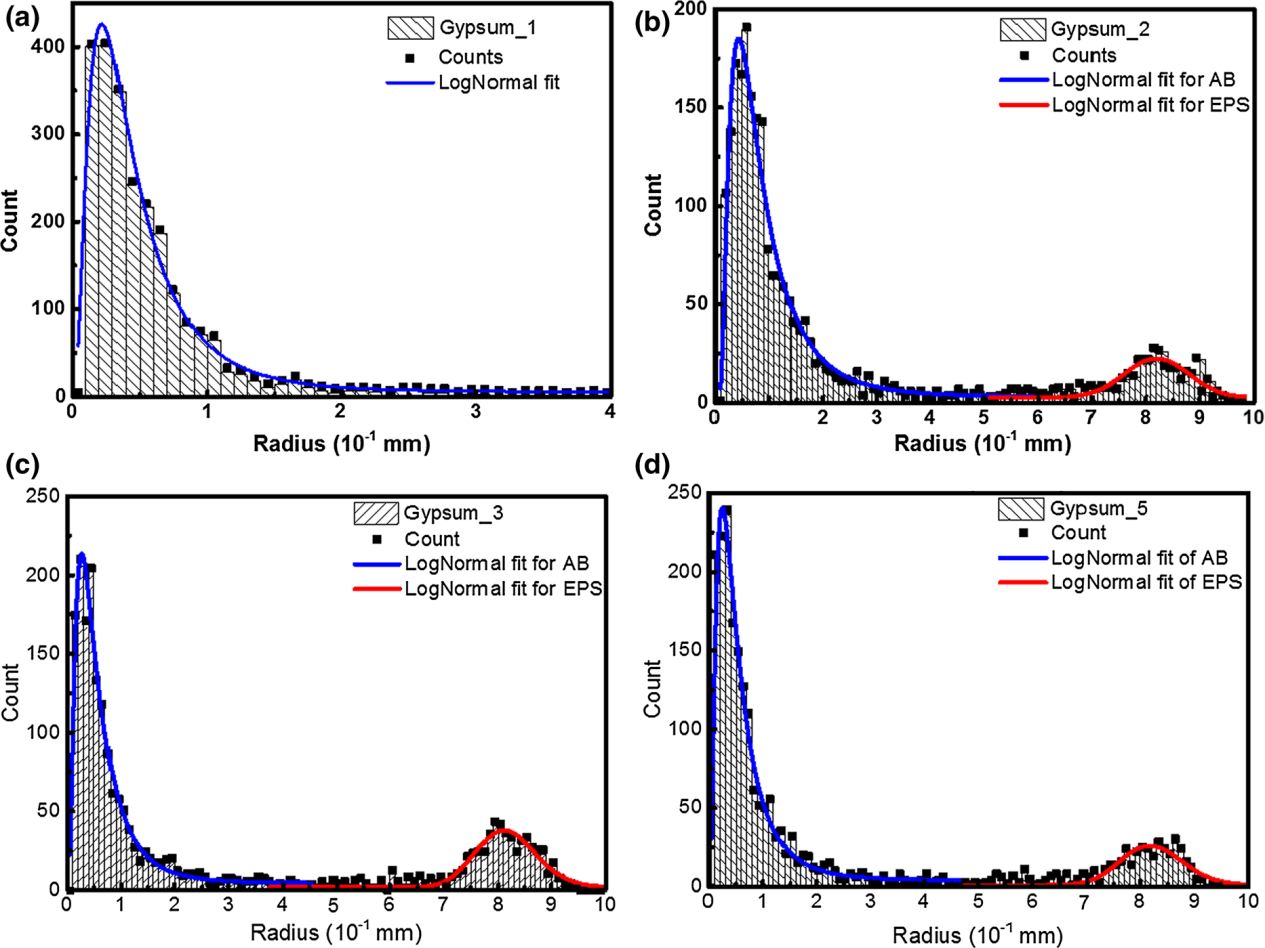
Distribution of the pores for specimens with **a** 0.5 vol%; **b** 12.94 vol%; **c** 22.56 vol%; **d** 30.41 vol% porosity. Note the additional distribution of pores between 0.7 and 1.0 mm radius. AB-air bubbles, EPS-polystyrene beads

The pores are quantified in terms of the equivalent radius, by converting the 3D volume to a sphere radius. Histogram distribution of the radii in four specimens are shown in Fig. 5. Two groups of pores can be clearly observed in the material. The first group consists of pores with a radius smaller than 0.5 mm, and these are considered to be air bubbles introduced during mixing or the curing process. The second group are the spherical expanded polystyrene beads added to the mixture with a radius ranging from 0.75 mm to 1.0 mm. For both group of pores, a log-normal function was found to best describe the size distribution - the fitting parameters for this relation between x (radius) and y (frequency) are listed in Table 1, where *A* is the area and *w* the standard deviation:

4 $$\begin{aligned} y=y_0 +\frac{A}{\sqrt{2\pi }wx}\cdot \frac{e^{\left( {-\ln \frac{x}{x_c }} \right) ^{2}}}{2w^{2}} \end{aligned}$$

**Table 1:** Fitting parameters for the log-normal distribution of the pores measured by tomography in the five specimens (Gyspum_1 to Gypsum_5)

	$$y_0 $$	$$x_c $$	*w*	*A*	$$\hbox {R}^{2}$$	$$\upchi ^{2}$$
Gypsum_1	0.12337	0.38962	0.74048	233.3309	0.98665	55.6
Gypsum_2_AB	2.03577	0.70599	0.75613	183.31226	0.97288	70.4
Gypsum_2_EPS	2.44122	8.31285	0.06594	27.17635	0.96421	10.7
Gypsum_3_AB	4.90982	0.48215	0.76369	143.46253	0.97539	77.3
Gypsum_3_EPS	2.47386	8.15369	0.06843	50.18796	0.89589	12.8
Gypsum_4_AB	6.18383	0.4124	0.71201	139.62279	0.98254	61.1
Gypsum_4_EPS	2.37848	8.25717	0.07071	46.30368	0.86439	19.7
Gypsum_5_AB	4.91943	0.47125	0.78047	159.50249	0.97899	80.7
Gypsum_5_EPS	1.94565	8.21656	0.07016	35.85676	0.89451	13.8

**Table 2:** The total number and volume percentages of the air bubbles (AB) and added polystyrene beads (EPS)

Sample number	Total porosity (vol%)	Total number of pores		Contribution to the total porosity (%)	
	AB+EPS	AB	EPS	AB	EPS
1	0.5	2342	0	100	0
2	12.9	2919	582	4.2	95.8
3	22.6	2640	959	2.8	97.2
4	30.4	3416	1290	2.7	97.3
5	30.0	4963	1292	3.4	96.6

The volume percentage of all the pores has been calculated from the tomography analysis to be 0.5, 12.9, 22.6, 30.4 and 30.0 vol%, respectively, for the five specimens tested (Table 2). The air bubbles (AB) usually have a radius less than 0.5 mm, Fig. 5a, as evident in the specimen with no added EPS. Radii of these air bubbles follow a log-normal distribution when no expanded polystyrene beads (EPS) are added to the mixture, Fig. 5. For specimens with added EPS, the number of the air bubbles and their volume have been calculated and listed in Table 2. The number of air bubbles is nearly constant for all specimens tested, except an increase in one specimen. However, regardless of the large number of air bubbles, their total volume accounts for less than 5% of the total porosity (Table 2). The EPS, on the other hand, form the main constituent of the total porosity, contributing more than 95% of pores in all the specimens measured. The modelling of the fracture characteristics of these materials focused mainly on the content of the added EPS.

The cubes ($20\times 20\times 20$ mm) used for X-ray tomography analyses are cut from the cast cube ($150\times 150\times 150$ mm) so the sides of the small cubes contain incomplete EPS pores. During the conversion from the 3D volume to the equivalent diameters for the measured pores, these incomplete EPS will show a smaller diameter compared to the intact EPS (Russ 2012). Therefore, it is possible that some of the small radius pores plotted in the histogram come from these ‘incomplete’ EPS. However, in Fig. 4 the range between the two main distributions are very low numbers, and this will not influence the calculation of the total porosity.

**Fig. 6:**
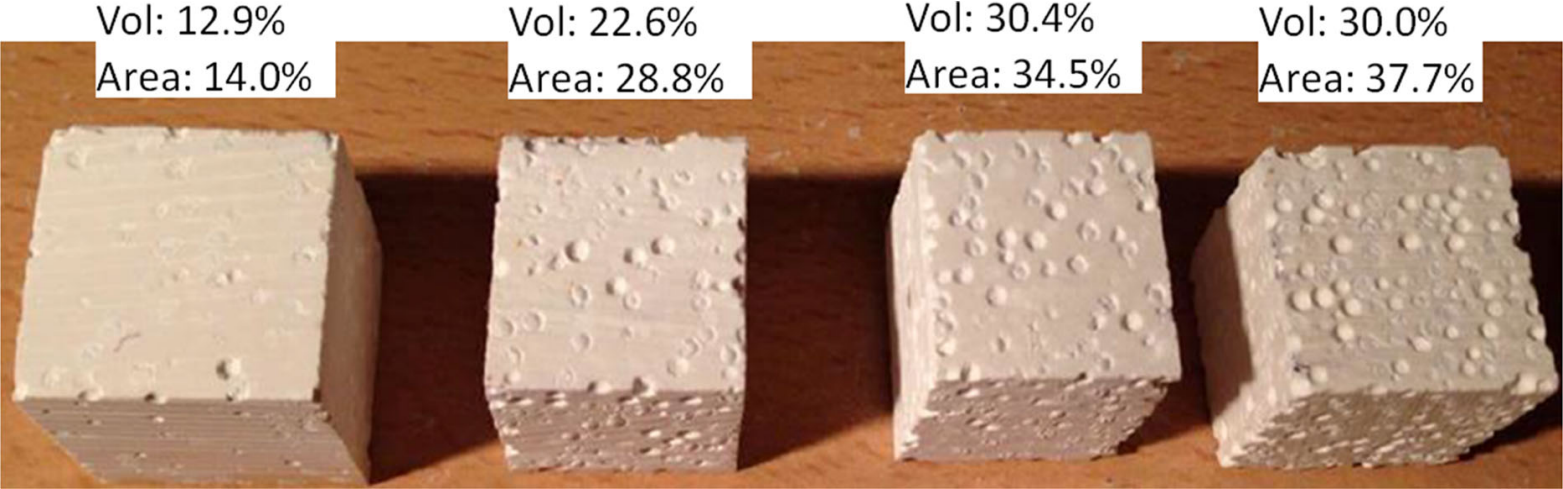
Four cubes (about $20\times 20\times 20$ mm) extracted from the large blocks representing 3D porosity in volume of 12.9, 22.6, 30.41 and 30.01%, respectively; the porosity was also measured in 2D in these individual cubes by optical microscope, and this give an evaluation of the porosity in 2D area ratio of 14, 28.8, 34.5 and 37.7%, respectively

In addition to the 3D tomography characterisation, 2D pore areas have been calculated. Optical imaging of the surface was undertaken (Fig. 6) and the area ratio of the pores have been manually picked and calculated using DpxViewPro software. By summing all pore areas for each surface and calculating the total surface area, a 2D porosity value is calculated to be 14.0, 28.8, 34.5 and 37.7%, for the cubes extracted from large samples with a 3D volume porosities of 12.9, 22.6, 30.4 and 31.7% respectively.

**Fig. 7:**
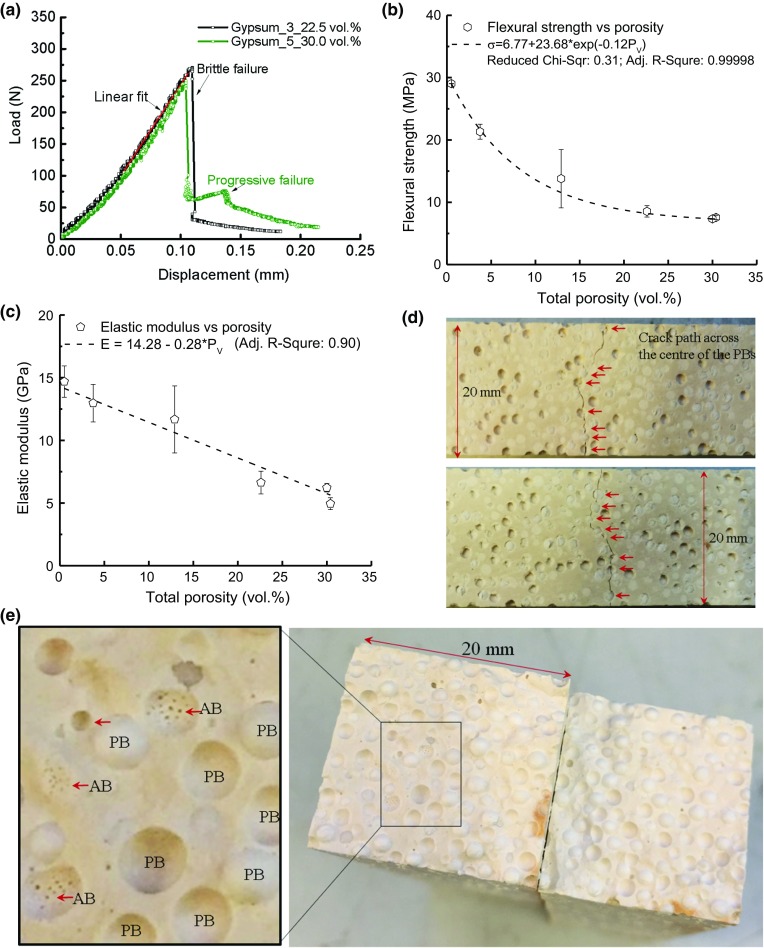
**a** Load-displacement curves for specimens with 22.5 and 30.0% respectively showing the transition between brittle fracture to progressive fracture with the increase of porosity; **b** the flexural strength decrease with porosity following an exponential decay; **c** the elastic modulus reduces linearly with increase porosity; **d** the tortuous crack paths created by four-point bending on the tensile surface of two specimens with 30.4 vol% porosity; **e** two fracture surfaces show the polystyrene beads (EPS) and the small air bubbles (ABs)

**Fig. 8:**
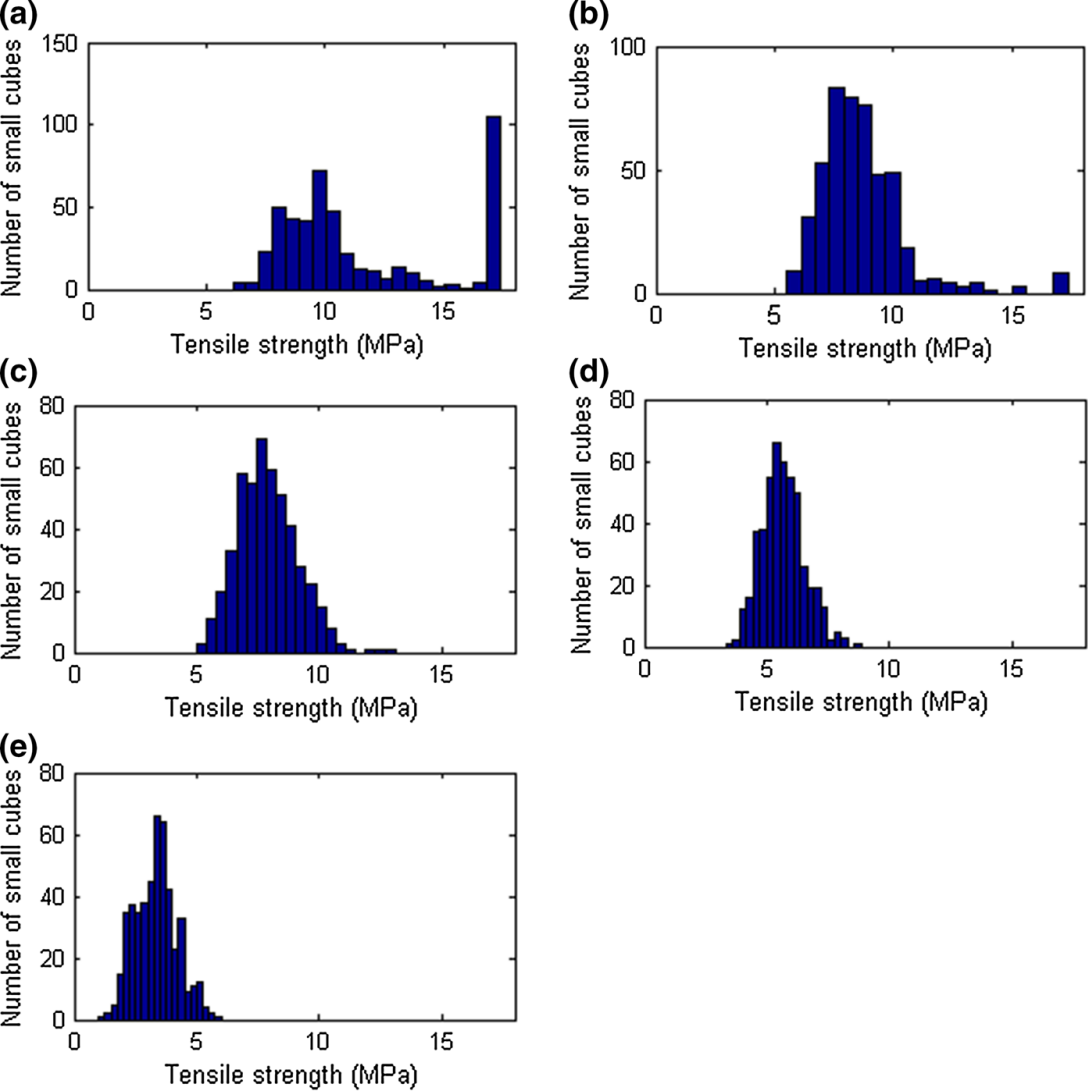
Distribution of simulated uniaxial tensile strengths of small ($$5\times 5\times 5$$ mm$$^{3})$$ cubes for different porosity levels. Porosities range from **a** 2%; **b** 5%; **c** 10%; **d** 20%; and **e** 40% porosity

### Mechanical properties

For the four-point bend tests, two typical load-displacement curves are shown in Fig. 7a. When the porosity is below 22.5 vol%, specimens show a ‘brittle’ fracture; whereas for specimens with a higher porosity, $\sim$30 vol%, a post-peak progressive failure was observed. Therefore, porosity promotes quasi-brittle behaviour. There is usually a bedding-in stage in the load-displacement curve, therefore, as shown in Fig. 7a, the elastic modulus was calculated using the linear gradient after this initial stage where the error introduced by the roller-specimen contact was considered minimum. Post-test examination demonstrated that no obvious crushing was observed for all the specimens tested. Therefore, it was considered that the roller-specimen contact has little influence on the modulus determination. The flexural strength and elastic modulus of the material was plotted as a function of the porosity in Fig. 7b, c, respectively. In general, there is a decrease from $\sim$29 to $\sim$7.3 MPa in the flexural strength as the porosity increases from 0.5 to 30.0 vol.%; the elastic modulus showed a similar reduction from 14.7 to 6.2 GPa.

Examination of the tensile surface of the fractured specimens showed that the cracks follow a path linking the added pores, Fig. 7d. Some of the specimens were loaded until the macro-crack propagated through the whole cross-section. An image of these fractured surfaces is shown in Fig. 7e also demonstrates that the fracture path followed the added porosity across the complete cross-section of the specimen. Some of the EPS remained attached to one of the fractured sections and were pulled out from the other half. This indicates that the interface between the EPS and the matrix was weak. An image with a higher magnification extracted from one part of the fractured surface, Fig. 7e, showed that there are clusters of small air bubbles attached to the interfaces between some of the EPS and the gypsum plaster.

### Modelling results

As stated previously, synthetic microstructures presented in Sect. 2.2.1 were sliced into $4\times 4\times 30$ small cubes (480 in total), each with a size of $5\times 5\times 5$ mm$^{3}$. Each small cube consisted of $20\times 20\times 20$ voxels, with a voxel size of 0.25 mm. As described in Sect. 2.2.2, these small cubes were then subjected to simulated uniaxial tensile testing. Depending on the porosity and pore size distribution within each small cube, the load displacement curve was determined. Clearly, these factors have an effect also on the uniaxial tensile strength of each small cube. Distributions of simulated uniaxial tensile strengths of small cubes for all simulated microstructures (i.e. porosity levels) are shown in Fig. 8.

**Fig. 9:**
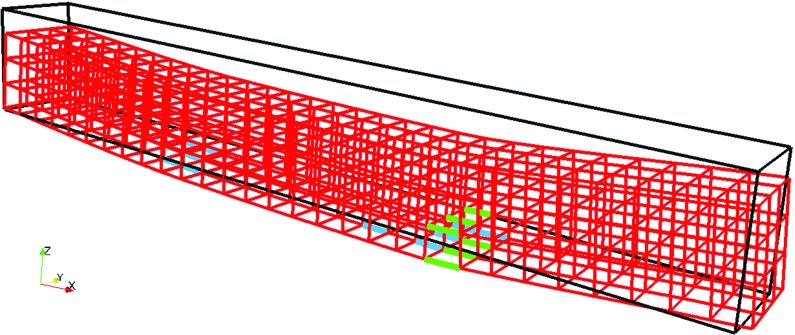
Deformed mesh at failure for the 20% porosity case loaded in the negative Z direction. *Green colour* denotes lattice elements that are completely damaged, while *blue colour* denotes lattice elements which are partially damaged. Deformations have been scaled for clarity

**Fig. 10:**
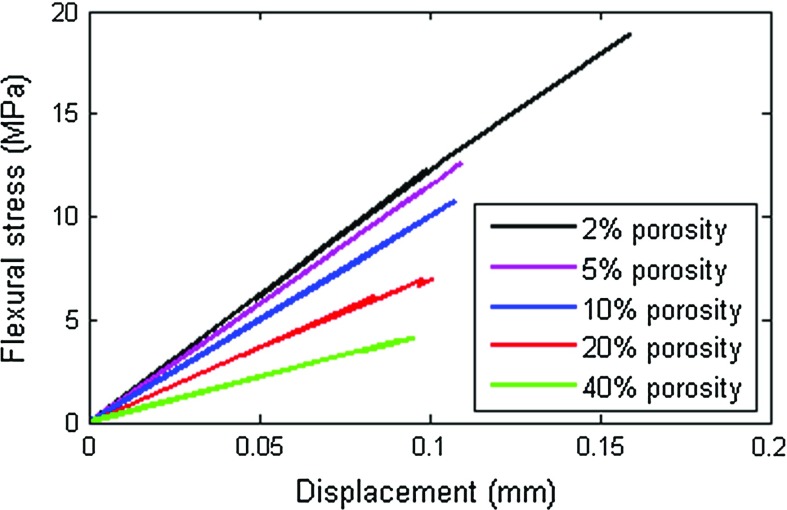
Simulated stress-displacement curves for gypsum plaster microstructures with different levels of porosity (1 per porosity level)

As described above, outputs of small-scale simulations were used as input for full-scale simulations. Use of numerical simulation has an advantage compared to experiments: in simulations, “specimens” can be tested multiple times. In order to investigate the scatter in simulated results, each microstructure was tested four times by loading it in four different ways. This was achieved by rotating the beam specimens around their longitudinal axis. In Table 3, all simulation results are summarized. Figure 9 shows a deformed mesh resulting from a full-scale simulation. Simulated stress-displacement curves for different levels of porosity are shown in Fig. 10.

**Table 3:** Summary of all simulation results (E—elastic modulus; $\hbox {f}_\mathrm{b}$—bending strength)

Loading direction	2% porosity		5% porosity		10% porosity		20% porosity		40% porosity	
	E (GPa)	$\hbox {f}_{\mathrm{b}}$ (MPa)	E (GPa)	$\hbox {f}_{\mathrm{b}}$ (MPa)	E (GPa)	$\hbox {f}_{\mathrm{b}}$ (MPa)	E (GPa)	$\hbox {f}_{\mathrm{b}}$ (MPa)	E (GPa)	$\hbox {f}_{\mathrm{b}}$ (MPa)
Y$+$	14.27	20.81	13.22	11.42	11.49	10.26	8.57	7.99	5.17	3.95
Y−	14.02	18.83	12.87	10.95	11.213	9.18	8.38	6.95	4.99	3.79
Z$+$	14.05	17.37	13.01	12.81	11.32	9.53	8.387	7.49	5.03	4.22
Z−	14.08	16.18	12.97	12.83	11.29	9.36	8.37	6.99	4.98	3.73
Average	14.11	18.30	13.02	12.37	11.33	9.58	8.43	7.35	5.05	3.92
Standard deviation	0.115	1.995	0.122	1.281	0.119	0.477	0.099	0.485	0.087	0.220

**Fig. 11:**
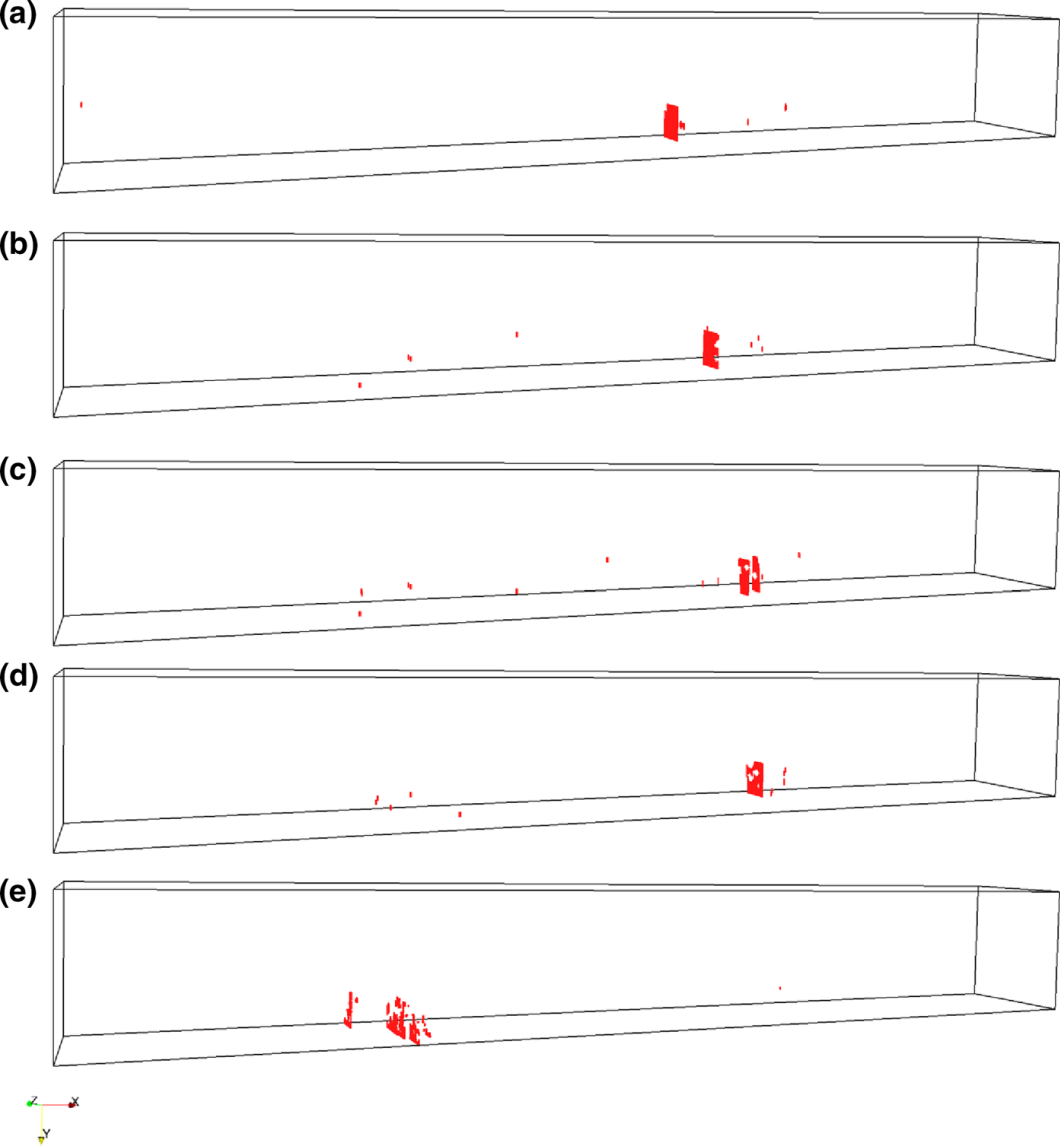
Microcracks (i.e. cracked beam elements) at peak load for simulations with different porosity levels (loaded in negative Y direction, i.e. from the *top*): **a** 2% porosity; **b** 5% porosity; **c** 10% porosity; **d** 20% porosity; and **e** 40% porosity

Apart from changing the global mechanical properties, increasing porosity also changes the fracture path, with cracking becoming more distributed as the porosity increases, Fig. 11. As stated previously, brittle fracture is characterized by a single crack, while quasi-brittle behaviour shows more distributed microcracks accompanying the “main” crack. A transition from brittle to more quasi-brittle behaviour is seen as the porosity levels increase, Fig. 11a, e.

**Fig. 12:**
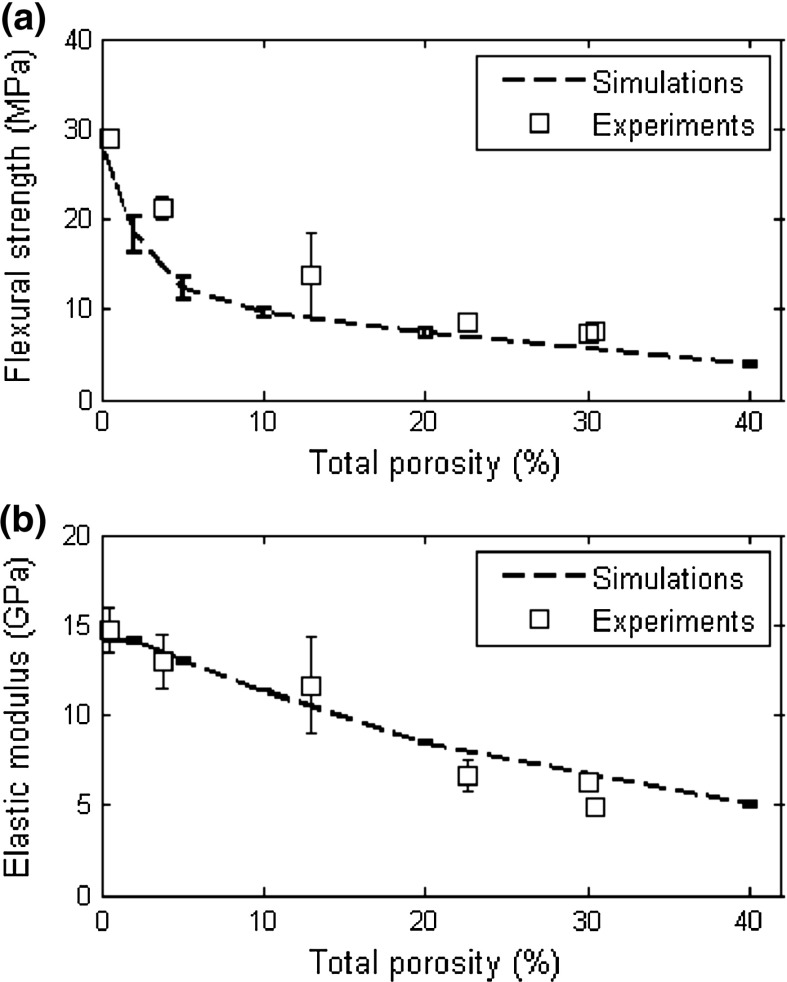
Comparison of the simulation results with experimental data for **a** flexural strength and **b** elastic modulus

In Fig. 12, simulated results are compared with the experimental values for flexural strength and the elastic modulus. Similar to the experiments, flexural strength decreases exponentially with increasing porosity. On the other hand, elastic modulus shows a linear decrease. Furthermore, the calculated values are quite close to the experimental values, i.e. within the experimental scatter.

## Discussion

In the past, EPS was mixed with cement to produce a material with reduced weight but improved thermal and hydro insulation properties (Al-Jabri et al. 2005). Most manufacturing processes involve either soaking the EPS in water for a period of time prior to mix with dry cement and aggregates, or use dry mixing but add a suspension agent when mixing with water. Due to hydrophobicity, these two methods contribute to a stronger interface between the EPS and the matrix material, hence a robust concrete. In the present case the EPS has been introduced into the gypsum as controlled volume fractions of spheres of known size distribution as a surrogate for porosity; therefore, a dry mixing process with no addition of suspension agents was used with the intention of producing a weak interface between the EPS spheres and the material matrix. It can be seen from the fractography analysis in Fig. 7 that cracks propagate to the middle of the EPS and result in the pull-out of the EPS as would have occurred for a pore. From the tomography measurements it is clear that a range of small air bubbles was produced during mixing, visually illustrated in Fig. 4 and statistically characterised in Fig. 5. However, these small air bubbles constitute only a small portion of the total pore volume (2.69% to 4.15%, Table 2). Indeed, pores reduce the actual volume of the material which carries the applied load, from which aspect, these small portion of small pores cause little error in the measured data. In addition, these small pores are observed even in the sample with no added EPS, and their volume and number remains nearly constant when the total porosity changes from 0 vol% (2324) to 22.5 vol% (2640; 2.78%). The total number increase in material with a higher content of EPS, but the percentage of the air bubbles stabilised at 2.69 and 3.39. Thus, the effect of small pores on the measured properties is negligible and the trend in the measured mechanical properties is primarily caused by the addition of surrogate porosity. It is worth pointing out that, since the EPS are solid, they can only simulate isolated closed pores with point touching. The packing of uniform and non-uniform diameter spheres has been studies extensively (Conway and Sloane 2013). As a consequence, ordered packing, including hombohedral and cubic arrangements, brackets the range of attainable porosities at 0.74 and 0.52 (Zhang et al. 2006). For random packing of the EPS, such as random close packing with no global ordering, the highest packing density (porosity) can be achieved is between 0.64 and 0.60 independent of the EPS size. Certainly, higher porosity could be achieved by introducing gap-grading, but the above limits bound the maximum porosity that can be simulated by using EPS with a narrow size range.

For the mechanical tests, the measured mechanical properties decrease with the increasing amount of surrogate porosity. For the flexural strength, there is an exponential decrease from 29 MPa in start condition to about 7.3 MPa for 30.4 vol% total porosity. For the elastic modulus, experiments show a more linear decrease (from 14.7 GPa to about 4.9 GPa) with increased porosity. It is not uncommon to see a linear change in modulus in materials with porosity, for example (Porter and Reed 1977) obtained a similar trend in refactory materials. Alternatively, Dorey et al. (2002) investigated the effect of pore clustering on the elastic modulus of ceramics and the value obtained in their study has shown a nearly linear shallow curve. In addition, the elastic modulus reduced by a smaller amount (66%) with porosity compared with strength (75%) within the same porosity range (0–30 vol.% total porosity)—this is consistent with the general observation that elastic modulus is less sensitive than strength for materials containing pores with one type of geometry. In terms of the shape of the load-displacement curves, Fig. 7a, the post-peak progressive failure was observed with higher EPS addition while for little or no EPS addition, it generally shows an abrupt failure, i.e. the failure mode changes from brittle to more quasi-brittle.

Similar to the experimental study, modelling results show a great impact of increased porosity on the simulated mechanical properties of gypsum plaster. Already at the small scale, an impact on uniaxial strength of simulated cubes is observed, Fig. 8. For the 2 vol% porosity, a large number of small cubes ($\sim$100) has an uniaxial tensile strength higher than 15 MPa, Fig. 8a; as stated previously, note that the input uniaxial tensile strength for a lattice element was 17.4 MPa. This suggests that a large number of small-cubes contain virtually no pores in this case. The remaining cubes show a distribution of uniaxial tensile strength ranging from $\sim$5 to $\sim$15 MPa, depending on their local porosity. For the specimen with 5 vol.% porosity, the number of small cubes without porosity is a lot smaller, Fig. 8b); for the 10, 20 and 40 vol% cases, there are no small-cubes which are pore free, which is reflected in a distribution of tensile strengths without the outliers, Fig. 8c, e.

Full-scale simulations show a trend consistent with the experiments: the decay of the elastic modulus displays a shallow curve and is approximately linear, Fig. 12b, while the decay of the flexural strength is exponential with increasing porosity, Fig. 12a. It is also interesting to observe that the scatter in the simulation results for the flexural strength and the elastic modulus is lower compared to the experimental results, Table 3 and Fig. 12. This is probably due to the nature of the simulations: for each simulated microstructure, four mechanical simulations are performed by rotating the specimen around the longitudinal axis. Therefore, four simulations are performed for four different loading directions, but the actual pore structure for each of them was kept constant. In experiments, however, several different specimens with potentially different pore structures resulted from the stochastic nature of the manufacturing process are tested. Hence, more scatter in the measurements are generated, and this is well illustrated by the flexural strength and the elastic modulus of the 12.5 vol% specimens, Fig. 12. In addition, a simulated material microstructure was used as input, and not the real microstructure. This results in a somewhat simplified description of the microstructure, and could be a source of differences between the experiments simulated values.

Representative stress-displacement curves, Fig. 10, show a clear decrease in strength and the elastic modulus with increasing porosity. Furthermore, the 2 vol% and the 5 vol% curves show very brittle behaviour with no post-peak, while 10, 20, and 40 vol% curves contain small post-peak deformations and quasi-brittle behaviour. Figure 11 illustrates that, in case of 2 vol%, only a small number of microcracks accompany the main crack; the number of microcracks increases with the porosity, while the 40 vol% specimen shows a tortuous crack path, Fig. 11d. This behaviour is observed also experimentally and shown in Fig. 7d.

## Conclusions

In this work, the influence of porosity on mechanical and fracture properties of quasi-brittle behaviour has been studied. A model material, based on hydrated calcium sulphate with added known amounts of porosity as expanded polystyrene spherical balls, was used as a media to investigate the evolution of microstructure and mechanical properties. Furthermore, numerical simulations using a microstructure-based multi-scale model were performed to replicate the experimental procedure. The fracture model uses only two mechanical parameters (fracture strength and the elastic modulus) and is able, together with the microstructural model, to correctly reproduce experimental observations. Based on the presented results, several main conclusions can be drawn:

- Addition of EPS spheres to gypsum plaster is a reliable way of simulating a brittle solid with controlled additions of porosity. A small fraction of air pores are present prior to the addition of surrogate porosity, which dominates the porosity and provides an ideal model material to consider deformation and fracture.
- In the model material, the measured elastic modulus decreases linearly with added porosity while the strength follows an exponential decay.
- Elastic modulus reduces less than the strength with increased porosity.
- Similar to the experiments, modelling results show a linear decrease of elastic modulus and an exponential decrease of flexural strength with increasing porosity. The values obtained are close to the experimental values, which provides confidence for use of the proposed model for porosities beyond experimentally measured, i.e. higher or lower porosity.
- The model has shown that the number of micro-cracks accompanying the main crack increases when more porosity is contained in the deformed volume. This signifies a transition from brittle to quasi-brittle behaviour with the increase in porosity.

It has to be noted that, in the current research, the mechanical model used a simulated material microstructure as input, as opposed to the real microstructure. This, of course, implies a simplified description of the microstructure, and causes some deviation between the experimental measurements and simulated values. In the future, current work will be expanded and material microstructures obtained by X-ray computed tomography will be directly used as input. This will provide even more confidence in predictive capabilities of the model, enabling its use in critical applications such as ageing management of nuclear and civil engineering structures.
